# Variations of health check attendance in later life: results from a British birth cohort study

**DOI:** 10.1186/s12889-019-7875-x

**Published:** 2019-11-28

**Authors:** Rebecca Wilson, Diana Kuh, Mai Stafford

**Affiliations:** 1 MRC Unit for Lifelong Health and Ageing at UCL, 1-19 Torrington Place, London, WC1E 7HB UK; 2 The Health Foundation, 8 Salisbury Square, London, EC4Y 8AP UK

**Keywords:** Health checks, Preventive health care, Screening, British cohort study, Longitudinal study

## Abstract

**Background:**

Older adults are advised to attend a number of preventive health checks to preserve health and identify risk factors for disease. Previous research has identified a number of health and social factors, labelled as predisposing, enabling and need factors, using Andersen’s Behavioural Model of Health Service Use, that are associated with health care utilisation. We aimed to assess associations between factors from childhood and adulthood, and health check attendance in later life in a British birth cohort study.

**Methods:**

For 2370 study members from the MRC National Survey of Health and Development (NSHD), health check attendance was assessed at age 68. Study members were asked if they: attended blood pressure and cholesterol checks, had their eyes tested, received the influenza vaccine, attended colon cancer screening and dental checks. Health and social factors from childhood and adulthood were used in binomial regression models to test associations with health check attendance in men and women.

**Results:**

Health check attendance was high; 41% reported attending all six health checks within the recommended time frame. In multivariable models, being a non-smoker and having more health conditions in adulthood were associated with greater health check attendance in men and women. In women, childhood socioeconomic advantage, being more physically active in midlife and previously attending screening procedures, and in men, greater self-organisation in adolescence and being married were associated with attending more health checks in later life, following adjustments for childhood and adulthood factors.

**Conclusions:**

A number of predisposing, enabling and need factors from childhood and adulthood were found to be associated with health check attendance at age 68, demonstrating the relevance of applying a life course perspective to Andersen’s model in investigating health check attendance in later life. Health related factors were found to be stronger correlates of health check attendance than socioeconomic factors.

## Background

Adults in the UK are advised to attend various preventive health services in later adulthood. These include general health checks to identify signs of or risk factors for particular diseases [1] and vaccination programmes offered to protect those most at risk from illness [2]. Certain preventive health checks and services are recommended to UK adults within specified timeframes. In England, the NHS Health Check programme aims to screen for cardiovascular risk factors in adults aged 40–74 every 5 years [3, 4]. Typically this will include a blood pressure and cholesterol check [5]. Furthermore, adults over age 65 are also advised to receive the influenza (flu) vaccine yearly [6], take part in colorectal cancer screening every 2 years [7] and attend eyesight and dental checks every 2 years [8, 9]. These preventive health care services will be collectively referred to as health checks from hereon in.

Engaging with preventive health checks can be a proactive way for older people to manage their health. They are voluntarily attended and may require asymptomatic individuals to take initiative to attend when the immediate need is not apparent. However, many older adults are managing several comorbid health conditions [10, 11] and accessing multiple health care services [12]. This could mean greater burden of health management for older adults and preventive health care may become less of a priority.

Previous research has reported potential benefits of health check and screening attendance, including lifestyle improvements [1], reduction in cardiovascular risk [13, 14], more diagnosis of hypertension and vascular disease [1, 14] and lower risk of premature mortality [15, 16]. Studies have reported that NHS Health Check uptake rates are between 20 and 48% [13, 17], although uptake of the flu vaccine is generally higher (70.5% of adults aged 65+ and 48.6% in clinical risk groups under 65 [2]). For colorectal cancer screening, around half (52%) of those offered a test engage with the programme [18].

Given that health checks and preventive health care are potentially beneficial to both patients and health care providers, by preserving health and financial resources [19], understanding factors associated with preventive health care engagement is important. Andersen’s Behavioural Model of Health Service Use [20] identifies three explanatory domains: predisposing characteristics (including demographics, social structure and health beliefs), enabling resources (personal/family and community factors) and need (individuals’ perceived need). Andersen [20] noted ‘discretionary’ health care services (in contrast with hospital attendance for more serious problems), such as dental services (and arguably other preventive health care checks), were more likely to be associated with certain predisposing factors and enabling resources. These include social structures (which includes socioeconomic factors, such as education and occupation), health beliefs, personal/family related factors and community resources.

A number of studies have utilised Andersen’s model when investigating health service use for a wide variety of health needs and service sectors, though studies differ in the factors examined [21]. A systematic review of studies using Andersen’s model reported that the most frequently researched variables measured as potential correlates of health care utilisation were age, marital status, gender, education, ethnicity, socioeconomic position (SEP), health status and having a family doctor [21]. The authors concluded that results were inconsistent regarding associations with health care utilisation. Studies of preventive health care utilisation suggest that attendance for health checks and screening services is associated with higher social class and higher levels of education [22–25], poor self-rated health [19], having previously accessed GP services [19, 23, 26], being a non-smoker [17, 19, 23] and being married [25, 26]. Findings for gender are mixed and may depend on the type of preventive health care being considered. Women are more likely to engage with colorectal screening [27], cardiovascular health checks [17, 28], blood pressure, cholesterol and dental checks [29]. Whilst European studies have shown men are more likely to receive the flu vaccine than women [30, 31], an American study reported the opposite [29]. Other research reported no gender differences in health check attendance [19].

Whilst several correlates of health check attendance have been identified, much of the previous research is cross-sectional. The study of life course epidemiology focuses on the long-term impact of exposures and experiences in childhood, adolescence and adulthood on later health and wellbeing [32]. A life course approach is relevant to the study of health check attendance, given that many health beliefs and lifestyle factors may be influenced by socioeconomic circumstances and experiences across child and adulthood [33–35]. There is a gap in the literature considering potential explanatory factors from earlier in life.

This study investigated whether health and socio-behavioural characteristics earlier in life were associated with health check attendance at age 68 in the MRC National Survey of Health and Development (NSHD). We hypothesised that various predisposing factors (socioeconomic advantage, and more healthy behaviours and family support), enabling factors (previous health care utilisation) and need factors (worse health across the life course) would be associated with greater engagement in preventive health care checks.

## Methods

The NSHD is the longest running British birth cohort study, which has collected data 24 times from a sample of 2547 women and 2815 men born in England, Scotland and Wales in 1 week in March 1946 from birth to the present day [36, 37]. Data have been collected using various data collection modes including face-to-face interviews, questionnaires and visits from health professionals. The sample remains representative of people born in mainland Britain in 1946 [38]. Data were most recently collected at age 68–69; 2638 study members completed a postal questionnaire and/or a home visit from a research nurse [39]. This represents 93.7% of the target sample (*N* = 2816 who remained in the study and were living in England, Scotland and Wales). Of the original sample (*N* = 5362), 957 had already died, 620 had previously withdrawn, 574 had emigrated and 395 had been untraceable for more than 5 years.

The sample included in this study comprised the 2370 study members living in mainland Britain and who completed the questionnaire at age 68 and had data for health check attendance measures (the main outcome used in this sample).

### Health check attendance

At age 68, study members were asked in the postal questionnaire about their attendance at a number of health checks recommended to people at that age. Questions were based on a subscale of a health risk appraisal instrument designed for use in older people [40]. Stuck and colleagues [40] developed and tested the instrument in England, Germany and Switzerland, with appropriate adaptations for regional differences in access to preventive care services. The preventive care subscale (which included checks for blood pressure and cholesterol, colon cancer screening and flu vaccination) was further adapted for use in NSHD (eyesight and dental checks were also added) based on recommendations for services in 2014, with regard to the recommended time frames within which to attend (recommended time frames are presented in Table 2).

Health check attendance was measured as a proportion; the number of health checks attended out of the possible 6. This variable ranged from 0 (where study members attended no health checks) to 1 (where study members attended 6 out of 6 checks). A summary measure of all health checks was used to capture engagement with preventive health care rather than to explore the predictors of individual health checks (which were not expected to vary [41]).

### Explanatory variables

*Predisposing factors* included gender, socioeconomic factors (namely SEP and educational attainment), psychosocial factors, health behaviours and family structure. Social class was measured using an ordered categorical variable based on fathers’ occupation at age 11 (or at age 15 or 4 if missing) and study members’ occupation at age 53, using the Registrar General’s social class classification, recoded from 1 to 6, where a low score indicated disadvantaged social class and a high score indicated more advantaged social class. Education was based on attainment by age 26; study members’ educational attainment was categorised as ‘no qualifications’, ‘lower-secondary’ (O-levels or equivalent), ‘advanced secondary’ (A-levels or equivalent) and ‘degree level’, and was measured using a categorical variable. Self-organisation in adolescence (capturing behaviours such as attitudes to work, concentration and neatness at school that previous research has shown are associated with health-related behaviours [42, 43]) was measured using a continuous scale (ranging from 1.0 to 5.0, where a higher score indicated a higher level of self-organisation) based on teachers’ ratings [44]. Smoking and physical activity were included as relevant health behaviours. Study members’ lifetime smoking behaviour by age 68 was classified from regular self-reports as lifelong non-smoker, ex-smoker or lifelong smoker. Physical activity in earlier adulthood was measured at age 43 and, if data were not available, at age 36; study members reported their participation in sports and leisure activities and were coded as either ‘inactive’, ‘less active’ if they participated in between 1 and 4 activities per month, or ‘most active’ if they participated in 5 or more activities per month (scored from low to high) [45]. Study members reported their marital status at age 68 as married, single, separated, divorced or widowed; separated, divorced and widowed were combined for analysis.

*Enabling factors* included measures of access to and utilisation of health care services. Study members reported if they had visited the GP for any reason in the last year and GP attendance in earlier adulthood was measured using a binary variable (not attended in the last year/attended in the last year) at ages 31 if available and 20 if not. Female study members reported if they had previously attended cervical screening at age 43 and a mammogram at age 53.

*Need factors* include measures of health status. Serious illness in early life was indicated if the study member had a hospitalisation lasting more than 28 days between the age of 0 and 25. The number of health conditions study members reported experiencing in the past 10 years (from a list of 15 health conditions including acute and chronic health problems) at age 53 measured health burden in midlife.

### Analysis

Binomial regression models were used to regress the proportion of health checks attended onto the explanatory variables described above. Preliminary analysis included gender by exposure interaction terms; likelihood ratio tests confirmed that gender interactions were evident for the following explanatory variables: childhood social class, education attainment, marital status and smoking behaviour. Further analyses were stratified by gender, also because different exposures were available for men and women.

Bivariate models were used to assess associations between each exposure and health check attendance (Model 1). For women, previous mammogram and cervical screening attendance were also included as potential explanatory variables. To explore the role of different life course exposures, multivariable models including all variables from childhood (Model 2) and from adulthood (Model 3) bivariately associated (at *p* < 0.05) with attendance were used. Finally, all variables associated bivariately with attendance were entered into a fully adjusted model (Model 4). A multivariable model (including all variables bivariately associated with attendance) was used to show if any associations were attenuated by other explanatory variables, either from childhood or adulthood. The sequential analysis adjusting for factors from childhood and adulthood was used to illustrate whether any factors from childhood were associated with health check attendance independently of factors from adulthood and if they were attenuated or not with the introduction of adulthood factors to the model.

Multiple imputation models using chained equations were used to impute missing data on explanatory variables in order to preserve the sample size for the analysis (*N* = 2370). Imputation models included all the covariates of interest and health check attendance. Five imputed datasets were created and estimates from regression models were combined using Rubin’s rules [46]. In sensitivity analyses, we also ran models using complete cases. No material differences were observed and so we present results based on imputed data only. All regression models (bivariate and multivariable) utilised imputed data if data were missing.

All analyses were done using Stata 14.

## Results

Sample descriptives are shown in Table 1. Missing data for all explanatory variables was below 11%.

**Table 1:** Predisposing, enabling and need factors described in the sample (*N* = 2370)

	N (%)
Predisposing factors	
Childhood social class	
Unskilled	122 (5.2)
Partly skilled	391 (16.5)
Skilled (manual)	695 (29.3)
Skilled (non-manual)	395 (16.7)
Intermediate	484 (20.4)
Professional	167 (7.1)
Missing	116 (4.9)
Adolescent self-organisation, mean (95% CI)	3.4 (3.4–3.4)
Missing, N (%)	247 (10.4)
Educational attainment at age 26	
No qualifications	738 (31.1)
Lower secondary	637 (26.9)
Advanced secondary	619 (26.1)
Degree level	252 (10.6)
Missing	124 (5.2)
Adult social class	
Unskilled	87 (3.7)
Partly skilled	240 (10.1)
Skilled (manual)	375 (15.8)
Skilled (non-manual)	562 (23.7)
Intermediate	909 (38.4)
Professional	177 (7.5)
Missing	20 (0.8)
Smoking status	
Never	663 (28.0)
Ex	1410 (59.5)
Current	137 (5.8)
Missing	160 (6.8)
Physical activity	
Inactive	1075 (45.4)
Less active	538 (22.7)
Most active	598 (25.2)
Missing	159 (6.7)
Marital status	
Single	86 (3.6)
Married	1760 (74.3)
Separated/divorced/ widowed	478 (20.2)
Missing	46 (1.9)
Enabling factors	
GP visits	
No previously reported GP visits	1111 (46.9)
Previously reported GP visits	1256 (53.0)
Missing	3 (0.1)
Ever attended mammogram age 53 (*N* = 1231 women)	
Never	60 (4.7)
Yes	1102 (89.5)
Missing	69 (5.6)
Cervical screening attendance age 43 (*N* = 1231 women)	
Attended + 5 years ago/never	130 (10.6)
Attended within last 5 years	1020 (82.9)
Missing	81 (6.6)
Need factors	
Childhood serious illness resulting in hospital admissions (age 0–25)	
No hospital admissions	1674 (70.6)
At least one hospital admission	696 (29.4)
Total number health conditions at age 53, mean (95% CI)	1.6 (1.5–1.6)
Missing	211 (8.9)

**Fig. 1:**
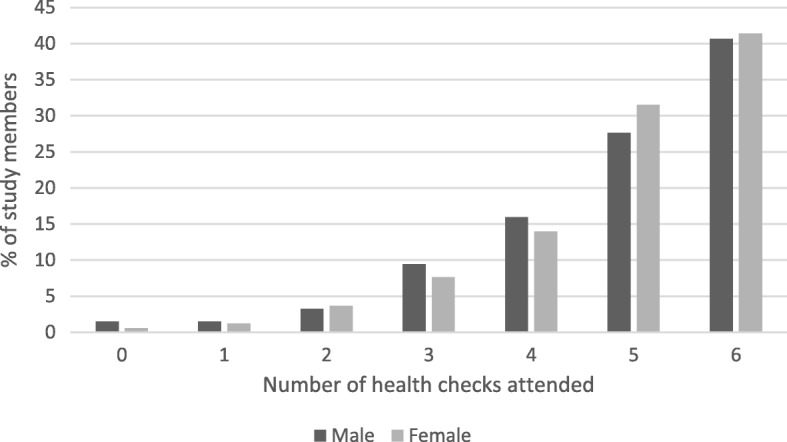
Number of health checks attended by male and female study members

Figure 1 shows the proportion of study members who reported attending 0–6 health checks within the recommended time frame at age 68. Attendance was high; 41.1% of study members reported attending all six health checks within the recommended time frame and 29.7% reported attending five health checks. No health checks were attended by 1% of study members.

Table 2 shows the percentage of study members who reported attending the health checks within the recommended timeframe. For every health check, attendance was above 73%. Blood pressure checks were the most frequently attended health check and the flu vaccine the least frequently attended, for both men and women. Attendance was higher in women for eyesight, dental checks, and for colon cancer screening; the greatest difference between men and women’s attendance was seen in eyesight checks (difference in attendance by 7%).

**Table 2:** Number of study members (%) that attended each health check

Health check (recommended frequency)	Total (*N* = 2370) N (%)	Men (*N* = 1139) N (%)	Women (*N* = 1231) N (%)
Blood pressure (within last year)	2169 (91.5)	1048 (92.0)	1121 (91.1)
Eyesight (within last two years)	2048 (86.4)	943 (82.8)	1105 (89.8)
Dental (within last year)	1960 (82.7)	913 (80.2)	1047 (85.1)
Cholesterol (within last five years)	1864 (78.7)	913 (80.2)	951 (77.3)
Colon cancer screen (within last two years)	1784 (75.3)	846 (74.3)	938 (76.2)
Influenza vaccine (within last year)	1746 (73.7)	833 (73.1)	913 (74.2)

In women, all predisposing factors, except marital status, were bivariately associated with health check attendance (Table 3, Model 1). Higher childhood social class, adolescent self-organisation, educational attainment and adult social class were all associated with higher health check attendance. Being a non- or ex-smoker, compared to being a current smoker, and being more physically active in midlife was associated with higher attendance. Of the enabling factors assessed, attending mammogram and cervical screening in midlife were both associated with higher attendance. GP attendance in earlier adulthood was not associated with attendance. Both need factors, measuring health earlier in life, were associated with attendance. A greater number of midlife health conditions was associated bivariately with higher health check attendance; in contrast serious illness earlier in life was associated with lower attendance.

**Table 3:** Table of associations between potential explanatory variables and health check attendance in women

Potential explanatory variable *N* = 1231		Model 1^a^ Coef (*p* value)	Model 2^b^ Coef (*p* value)	Model 3^c^ Coef (*p* value)	Model 4^d^ Coef (*p* value)
Predisposing factors					
Childhood social class (per one class increase; unskilled ➔ professional)		***0.16 (< 0.01)***	***0.15 (< 0.01)***		***0.09 (0.02)***
Adolescent self-organisation (per one unit increase in the self-organisation factor score)		***0.18 (< 0.01)***	0.13 (0.06)		0.09 (0.23)
Educational attainment at age 26 (No qualifications used as reference group)	Lower secondary	***0.48 (< 0.01)***		***0.34 (< 0.01)***	***0.25 (0.01)***
	Advanced secondary	***0.58 (< 0.01)***		***0.35 (< 0.01)***	0.19 (0.13)
	Degree level	0.34 (0.06)		0.03 (0.89)	−0.23 (0.31)
Adult social class (per one class increase; unskilled ➔ professional)		***0.14 (< 0.01)***		0.06 (0.08)	0.06 (0.12)
Smoking behaviour to age 68 (Current smoker used as reference group)	Never smoked	***0.73 (< 0.01)***		***0.43 (0.02)***	***0.40 (0.03)***
	Ex-smoker	***0.76 (< 0.01)***		***0.51 (< 0.01)***	***0.49 (< 0.01)***
Level of physical activity in adulthood (per one level increase in activity; inactive ➔ most active)		***0.19 (< 0.01)***		***0.12 (0.02)***	***0.11 (0.03)***
Marital status age 68 (married used as reference group)	Single	−0.07 (0.70)			
	Separated/Divorced/Widowed	−0.19 (0.05)			
Enabling factors					
Any GP visits in earlier adulthood (compared to none)		0.13 (0.11)			
Mammogram attendance in midlife		***1.04 (< 0.01)***		***0.79 (< 0.01)***	***0.76 (< 0.01)***
Cervical screening attendance in midlife		***0.51 (< 0.01)***		***0.32 (< 0.01)***	***0.32 (< 0.01)***
Need factors					
Childhood serious illness resulting in hospital admissions (age 0–25) (compared to none)		***−0.20 (0.03)***	−0.18 (0.05)		−0.17 (0.06)
Total number of health conditions between ages 43–53 (per one condition increase)		***0.10 (< 0.01)***		***0.09 (< 0.01)***	***0.09 (< 0.01)***

^a^Bivariate associations

^b^Adjusted for all variables from childhood associated bivariately (*p < 0.05*) with health check attendance

^c^Adjusted for all variables from adulthood associated bivariately (*p < 0.05*) with health check attendance

^d^Adjusted for all variables from both childhood and adulthood associated bivariately (*p < 0.05*) with health check attendance

All bold values are statistically significant (*p* < 0.05)

Factors from childhood (social class, self-organisation and serious illness) were adjusted for in a multivariable model (Table 3, Model 2). Associations between self-organisation and serious illness and attendance were somewhat attenuated by childhood social class, higher social class remaining associated with higher attendance.

Model 3 (Table 3) included all factors from adulthood associated bivariately with health check attendance (educational attainment, social class, smoking behaviour, physical activity, mammogram and cervical screening attendance and number of health conditions in midlife). Most associations remained significant in the adjusted model, apart from the association between being educated to degree level, compared to having no qualifications, and higher attendance, which was attenuated by other health and social factors from adulthood. The association between adult social class and attendance was also attenuated.

In a fully adjusted model (Table 3, Model 4), higher childhood social class, having a lower-secondary level education (compared to having no qualifications), being a non- or ex-smoker (compared to being a current smoker), more physical activity in midlife, previously attending mammogram and cervical screening and worse health in midlife were significantly associated with higher health check attendance. Following adjustments, serious illness early in life was a weak correlate of lower attendance (*p* = 0.06). Associations between higher levels of education (advanced secondary and degree level) and attendance were fully attenuated, as was the association between higher adult social class and higher attendance.

In men, predisposing factors associated bivariately (Model 1, Table 4) with higher health check attendance were: higher adolescent self-organisation, higher educational attainment (being educated to advanced secondary or degree level, compared to having no qualifications), higher adult social class, and being a non- or ex-smoker (compared to being a current smoker). Being single or separated/divorced/widowed, compared to being married, was associated with lower health check attendance. Reporting more health conditions in midlife, but not serious illness earlier in life, was the only need factor associated with higher attendance. Childhood social class, physical activity in earlier adulthood and visiting a GP were not associated with health check attendance in men. Following adjustments for factors from adulthood bivariately (Model 3, Table 4) associated with attendance, the association with adult social class was attenuated and, of the associations between higher educational attainment and higher attendance, only the association between degree level education and higher attendance remained statistically significant.

**Table 4:** Table of associations between potential explanatory variables and health check attendance in men*

Potential explanatory variable *N* = 1139		Model 1^a^ Coef (*p* value)	Model 3^b^ Coef (*p* value)	Model 4^c^ Coef (*p* value)
Predisposing factors				
Childhood social class (per one class increase; unskilled ➔ professional)		0.04 (0.18)		
Adolescent self-organisation (per one unit increase in the self-organisation factor score)		***0.26 (< 0.01)***		***0.18 (< 0.01)***
Educational attainment at age 26 (No qualifications used as reference group)	Lower secondary	0.13 (0.29)	−0.03 (0.84)	−0.01 (0.95)
	Advanced secondary	***0.29 (< 0.01)***	0.20 (0.08)	0.14 (0.25)
	Degree level	***0.52 (< 0.01)***	***0.40 ( 0.01)***	0.25 (0.15)
Adult social class (per one class increase; unskilled ➔ professional)		***0.12 (< 0.01)***	0.03 (0.40)	0.03 (0.54)
Smoking behaviour to age 68 (Current smoker used as reference group)	Never smoked	***0.51 (< 0.01)***	***0.31 (0.04)***	0.28 (0.07)
	Ex-smoker	***0.54 (< 0.01)***	***0.36 (< 0.01)***	***0.35 (< 0.01)***
Level of physical activity in adulthood (per one level increase in activity; inactive ➔ most active)		0.07 (0.15)		
Marital status age 68 (Married used as reference group)	Single	***−0.44 (0.05)***	−0.42 (0.06)	−0.44 (0.05)
	Separated/ Divorced/ Widowed	***−0.51 (< 0.01)***	***− 0.48 (< 0.01)***	***−0.47 (< 0.01)***
Enabling factors				
Any GP visits in earlier adulthood (compared to none)		0.14 (0.10)		
Need factors				
Childhood serious illness resulting in hospital admissions (age 0–25) (compared to none)		−0.08 (0.35)		
Total number of health conditions between ages 43–53 (per one condition increase)		***0.17 (< 0.01)***	***0.16 (< 0.01)***	***0.17 (< 0.01)***

*Model 2 is omitted as only one variable from childhood (self-organisation) was associated with health check attendance

^a^Bivariate associations

^b^Adjusted for all variables from adulthood associated bivariately (*p < 0.05*) with health check attendance

^c^Adjusted for all variables from both childhood and adulthood associated bivariately (*p < 0.05*) with health check attendance

All bold values are statistically significant (*p* < 0.05)

In a fully adjusted model, including self-organisation and variables from adulthood bivariately associated with health check attendance (educational attainment, social class, smoking behaviour, marital status and number of health conditions reported in midlife; Model 4), higher adolescent self-organisation, being an ex-smoker (compared to a current smoker) and reporting more health conditions in midlife were associated with higher attendance and being separated/divorced/widowed (compared to being married) remained associated with lower attendance.

Several differences between men and women were observed. More advantaged childhood social class was associated with higher attendance in women following adjustments for childhood and adulthood factors, whereas in men, no association was found. For men, higher adolescent self-organisation was associated with higher attendance, also after adjusting for childhood and adulthood factors, whereas this was not observed in women. The association between higher educational attainment and higher attendance was fully attenuated in men, whereas in women, there was only partial attenuation. Being separated/divorced/widowed for men was strongly associated with lower attendance in the fully adjusted model, whereas this was not observed in women.

## Discussion

Predisposing, enabling and need factors were found to be associated with health check attendance in early old age, suggesting that all elements of Andersen’s model [20] are relevant. Furthermore, factors from both childhood and adulthood were associated with attendance, demonstrating the importance of a life course approach.

Factors that were associated with health check attendance in both men and women were identified. Smoking, a predisposing factor, was associated with lower attendance, after all adjustments. The association between not currently smoking and higher attendance was in line with previous research [17, 19, 23, 28]. Worse health in adulthood (a need factor) was associated with higher attendance in men and women, also supported by previous research [19, 26, 28]. In fully adjusted models, we demonstrated that these two measures of health and health behaviour were associated with health check attendance independent of social factors, by which these associations might operate.

More differences than similarities between men and women were observed. In men, higher adolescent self-organisation was associated with higher health check attendance, after adjusting for health and social factors from adulthood. This demonstrated evidence that this factor was associated with attendance independent of (i) social factors from adulthood and (ii) health behaviours (that previous research has shown are associated with this construct [42, 43]). Physical activity was significantly associated with attendance only in women; this remained a significant correlate in the final model.

Social factors from childhood and adulthood were associated with attendance. Differences were observed between men and women. In men, bivariate associations between social class and education and attendance were attenuated by measures of health and health behaviours. Whereas, in women, higher childhood social class was associated with higher attendance after adjusting for other childhood and adulthood factors, whilst adulthood social class was largely attenuated in the fully adjusted model. A weak association between education and attendance was observed in women. The association between the highest levels of education and higher attendance was attenuated; this may reflect the generally lower level of education in women, compared to men, in this British generation. Associations found between more advantaged social class and greater educational attainment and attending health checks were in line with previous studies [23, 26].

Being single or separated/divorced/widowed, compared to being married, was associated with lower attendance, after adjusting other factors. This association was observed only in men, suggesting that marriage has more of a role in health care attendance in men than it does in women; this gender difference has been reported in a previous study [47]. In women, attending mammogram and cervical screening – preventive health care services - in midlife were both associated with higher attendance. This suggests that earlier attendance to preventive health care services might be linked with the continued engagement with preventive care into later life.

By sequentially adjusting for factors from childhood and adulthood we observed that, whilst some associations between childhood exposures and health check attendance in later life were attenuated by factors from adulthood, associations remained between certain childhood factors and attendance, following adjustment for factors from adulthood: childhood social class in women, and self-organisation in men. Childhood factors that were attenuated by adulthood factors were self-organisation and child health in women.

Though most results supported our hypotheses, we were presented with some unexpected findings. Novel findings included the association between higher adolescent self-organisation and men’s higher attendance. It was expected that this association would be attenuated by either higher educational attainment or better health behaviours in adulthood (previous research has found higher self-organisation predictive of healthier behaviours [42, 43]), yet this was not so. However, as a teacher-rated measure, this variable may have limitations. It is possible that this association could be explained by an external factor not measured in the present study, or these results could suggest that an individuals’ level of self-organisation (a construct capturing behaviours such as work-related attitudes and concentration) in earlier life persists throughout adult life and is associated with proactive management of health. The associations found in women between social class, health and attendance were surprising. The association between higher adult social class and higher attendance was largely attenuated, whilst the association between childhood social class and attendance – in the same direction - remained following adjustments for health and social factors from adulthood. These results would suggest that the socioeconomic circumstances a woman is exposed to in her earlier life has a lasting effect on her health-related attitudes and behaviours later in life. The measure of social class in adulthood used in the study was a measure of the study members’ occupation, not that of the head of the household. This may have implications for this generation of women, who generally had lower levels of education and possibly were less likely to have higher occupational positions during their working life. This might reflect a discrepancy between the measures of social class in childhood (which captured the child’s father’s occupation) and in adulthood, which could explain the difference between the two associations. Furthermore, in women, we found an association between worse health in childhood and lower attendance (albeit weaker after adjustments) and an opposite association between worse health in adulthood and higher attendance. Although it could be argued that different measures were used (hospitalisations due to serious illness in childhood compared with number of health conditions in midlife), these results add to the existing evidence base by taking a life course perspective, demonstrating that exposures and experiences at various times across the life course may be associated differently with health care outcomes. The interplay between health and social factors throughout childhood and adulthood is likely to influence the way various experiences and exposures (including health states) affect how an individual manages their health. For example, in childhood, an individual’s health is primarily cared for by a parent, caregiver or health professional, whereas in adulthood, the majority of health challenges are ‘self-managed’ by an individual, or a health professional when needed. Our results show the relevance of a life course approach and highlight the benefit of exploring associations with both childhood and adult factors.

Health check attendance was higher than anticipated in this sample; recent studies have reported NHS Health Check (which generally includes blood pressure and cholesterol checks) uptake rates of between 20 and 48% [13, 17], however these statistics apply to those individuals invited to attend the NHS Health Checks, which includes adults over the age of 40, and is not directly comparable with the NSHD sample at age 68; it may be that engagement with preventive health care increases in older age. Rates of colorectal cancer screening was higher in this sample (75.3%) than the general English population (52%) [18], as was flu vaccine uptake; 73.7%, compared with 70.5% in the general population [2]. These results would suggest that members of a birth cohort study may be more health aware, both in terms of their own health and potential health challenges and of available and recommended preventive health care services. However, despite the higher uptake of health checks in this study, the associations between exposures and health checks should not be affected. Attitudes towards health checks have not always been positive: Krogsbøll et al. [1] described some of the possible risks from health check attendance including over-diagnosis and over-treatment, the implications of false positive and false negative results and adverse effects of invasive follow-up tests. These have been cited in the past as reasons for low attendance, however in this sample, low attendance was not observed.

### Strengths and limitations

The strength of the present study is that we utilised prospective, longitudinal data from a nationally representative cohort study, which allowed us to examine the correlates of health check attendance from across childhood and adulthood, whereas previous studies predominantly included concurrent correlates of health check attendance in adulthood. However, one limitation, mentioned above, was that health check attendance was high in this sample. Furthermore, several potential covariates were omitted from analysis, particularly those regarding locality, as previous literature has shown that NHS Health Check coverage differs by location and between different primary care practices [4] and factors that might hinder access to services, such as car ownership. However, we were able to represent each of the three domains (predisposing, enabling and need) from Andersen’s model [20], although we acknowledge that, due to the availability of data, certain areas were under-represented (particularly enabling factors and needs factors). This echoes the conclusion drawn by a review of studies utilising the same model; relevant variables from Andersen’s model are often missed in secondary analysis, due to the availability of data.

### Implications

Our findings have implications for health care providers and policy makers who may wish to target certain groups in order to improve health check attendance. The results from this study would suggest that certain groups could be targeted to improve health check attendance, including current smokers, women who are physically inactive, men who are not married and people with fewer reported health conditions in midlife. Furthermore, interventions to encourage health check attendance could begin targeting people in earlier adulthood, as our results would suggest that people who are engaged in preventive health care services in earlier adulthood are more likely to attend health checks in later life. As health checks are intended to both preserve the health of the older population and financial resources [19] and are funded for and made available to older adults, it is important to identify groups that are less likely to engage and to encourage attendance.

## Conclusions

In conclusion, having more health conditions in midlife and not smoking were associated with higher health check attendance in men and women, after adjusting for a number of factors from childhood and adulthood. In men, adolescent self-organisation and being married were also associated with attendance and in women, childhood social class and physical activity and previously attending screening procedures were associated with attendance after adjusting for other adult factors. Overall, there is more support for associations between health-related factors and attendance than socioeconomic circumstances (social class and education) and attendance. These results also demonstrate how predisposing factors from across life are associated with attendance in later life and varied between men and women, highlighting the importance of a life course approach when investigating engagement with preventive health care.

## Data Availability

Data used in this publication are available to bona fide researchers upon request to the NSHD Data Sharing Committee via a standard application procedure. Further details can be found at http://www.nshd.mrc.ac.uk/data. doi:10.5522/NSHD/Q101; doi:10.5522/NSHD/Q102; 10.5522/NSHD/Q103

## Abbreviations

NSHD: National Survey of Health and Development
SEP: Socioeconomic position
